# Accountability Metrics and Paying for Performance in Education and Health Care

**Published:** 2010-08-15

**Authors:** John F. Witte

**Affiliations:** University of Wisconsin-Madison

## Abstract

The track record in paying for performance in education is not good; nevertheless, emphasis on accountability and performance has gained momentum in the last 25 years. This emphasis includes systems of merit pay, career ladders, and national board certification. The general failures of these efforts have led some reformers to suggest that teacher pay be directly related to student value-added performance. This suggestion remains controversial but is also the hottest topic in paying for performance in education. Although many similarities exist between education and health care, major differences may make it even harder to install pay-for-performance systems in health than in education. If those systems are to be tried, experiments should begin in a bottom-up fashion at the unit level, rather than being imposed systemwide.

## Introduction

The track record in paying for performance in education is not good; nevertheless, the issue has gained momentum in the last 25 years. Although education and health care share several similarities — for example, both are professionally labor-intensive and have flatter hierarchies than other fields — their differences may make installing pay-for-performance systems more difficult in health care than in education. Because of the amount of money spent on each of these fields and their role in society, however, even small changes that enhance performance and accountability will yield considerable benefit.

In each field, system-level incentives should be distinguished from individual-level incentives. In education, the system levels are the district or school, and the individual levels are primarily teachers. In health care, the system levels would be units such as clinics or hospitals and departments within those units. The individual levels would be caregivers, including doctors, nurse practitioners, physician assistants, nurses, and aides. Accountability and incentive systems at the system level would differ from those at the individual level.

This article summarizes accountability, performance metrics, and reward systems in education for possible use in health care. First, I describe advances in education, emphasizing changes in accountability and achievement measures. Second, I review salary systems and individual- and system-level incentive and accountability efforts in education. Finally, I discuss the implications for health care of these efforts in education.

## Changing Emphasis on Student Achievement and Institutional Performance

The modern era in accountability in American education approximately dates from the publication of *A Nation at Risk* (1) in 1983. That national report was a scathing attack on the quality and competitiveness of American schools. Before that time, emphasis on student achievement or achievement-based accountability was lacking. Instead, emphasis was on education inputs and equity in resources.

That emphasis began to change, first through state actions, often led by governors, and later by the 2002 reauthorization of the Elementary and Secondary Education Act, known as No Child Left Behind (NCLB). Today, all states have achievement test score data in multiple subjects in grades 3 through 8 and 1 grade in high school. Data on grade retention and high school graduation are vastly improved. In some states, administrators and researchers can follow the achievement progress of individual students, allowing study of education growth from grade to grade. As required by NCLB, data are also made available to the public on the achievement performance of individual schools and districts. For schools that fail to meet performance standards, sanctions can be imposed. These changes amount to a revolution in terms of data, data availability, and a shift from a focus on education inputs to student outcomes. They also provide the potential for institutional and teacher accountability.

## Salary Systems, Paying for Performance, and Other Reward Efforts in Education

Methods of paying teachers have evolved over time. In the 19th century, education was generally limited to children of affluent families and took place in students' homes. Teachers were paid in room and board and often migrated from home to home (2). As schools became widespread in the mid-19th century, salaries were often arbitrarily based on sex, education, and the grade of the classroom. The inequities of differentiated salary scales between men and women were the target of emerging teachers' unions and women's equality movements. The result was the single-salary schedule first adopted in 1921. This schedule applied to all teachers and was based solely on years of experience and the teacher's education. The idea caught on quickly and, by 1950, 97% of school districts had adopted the single-salary schedule (2). Although it persists as the foundation of teacher compensation in public schools today, attempts to build incentives for performance have been proposed repeatedly in the last 3 decades. An underlying difficulty is that people disagree over what defines performance.

### Merit pay

The idea of merit pay has gained considerable attention during the past 25 years, although little systematic research shows the effects of merit pay on student achievement. Existing research suggests somewhat negative effects; however, these studies have several problems. First, "merit pay" encompasses a range of approaches to teacher evaluation and reward, but most merit pay rewards come either as one-time bonuses or as advances on the salary scale (3). Second, unmeasured selection problems may exist, both in terms of teachers, where missing variables may be the real driver of results, and for students, who may be nonrandomly assigned to teachers. Third, the best estimate of the number of public districts at any given time that are participating in some form of merit pay is 10% to 15% (3,4). Finally, merit pay plans do not last long in school districts. Of the plans in existence in 1983, 75% were gone by 1993 (5). In a study by Ballou, only approximately 25% of merit pay plans survived during a 6-year period (3).

Two reasons explain why merit pay plans in education do not persist. First, the characteristics of teaching make assessment of and support for incentive pay plans difficult, if not impossible. The art of teaching is hard to translate into objective measures and is a joint product of many people, and the links between teaching and student achievement are elusive (6). Second, teachers' unions oppose merit pay (4). One study compared pay-for-performance systems in public and private schools by using data from the national Schools and Staffing Surveys for a 6-year period (3). The percentages of schools and districts with merit pay plans were approximately the same in public and private sectors. However, that was driven by Catholic schools, which represented more than half of private schools. For the most recent year of the data (1993), the percentages of districts or schools with some form of merit pay plan were public, 12%; Catholic, 10%; other religious, 21%; and nonsectarian private, 35% (3). Catholic schools may have been under resource constraints, but other private schools demonstrated that merit pay plans could exist in high numbers. Public schools with collective bargaining agreements (64% of schools surveyed) had considerably fewer merit pay plans and lower plan survival rates than did schools with a "meet and confer system" (7% of schools) or that had no unions (29% of schools). The proportion of salary attributed to merit pay was 0% for schools under collective bargaining but 4% for schools with no union (3). Thus, the union environment affected the creation, longevity, and effect of merit pay plans.

The reasons for union opposition have, in part, to do with disagreement over what constitutes high performance and how it should be measured. The Obama Administration, in "Race to the Top" funding competitions, stresses the need to use student achievement test data as part of merit pay systems. Because unions often resist these methods, surely as the exclusive definition of meritorious performance, many unions refused to sign off on state proposals, and some states refused to apply at all.

### Career ladders and national board certification

Another approach to incentives in education has been to try to define certification categories. Since teachers traditionally are either probationary or not, the only route to advancement is to leave teaching and become an administrator. To alleviate this problem and to reward successful teachers, states and districts have created various career ladder opportunities. Career ladder systems differ in terms of how the ladders are set up, how teachers advance, and what rewards they receive. Beginning in 1987, a national board certification process was established for individual teachers.

As with merit pay, only recently have rigorous, empiric studies assessed the effects of these programs on student achievement. One of the best studies was of the Tennessee Career Ladder Evaluation System, which began in 1985 (7). The system was rigorous in terms of evaluative criteria and standards. The design of the program included consequential rewards; moving from probationary status to the third (top) rung of the ladder could add up to $10,000 to a teacher's base salary. Teachers moved up after extensive evaluations by principals and state officials.

However, as with merit pay studies, investigators found mixed results on achievement. One study found that having a teacher on a higher rung of the career ladder increased achievement in math but not reading (7). However, that result was confined to teachers only at the first of 3 possible rungs of the ladder. Equally problematic was a program audit that found that 95% of those who attempted that rung were given the certificate; 69% of teachers were on the first rung, and only 7% in were on rungs 2 or 3. After 2 years, the program was made voluntary, and it was terminated by the state legislature in 1997 because of a lack of funds.

The only national-level career development system is a certification process begun in 1987 by the National Board of Professional Teacher Standards (NBPTS). That process, which is voluntary for teachers, allows national certification after a screening and assessment process that includes construction of teaching portfolios (including video recordings of instruction); evidence provided by students, parents, and colleagues; and assessments of teaching practices, methods, and pedagogy. The process usually takes several years. Many states provide application grants and monetary rewards for completing the certification process.

Three major studies have assessed the effects of NBPTS certification on student achievement and teacher effectiveness. Two studies in North Carolina found varying degrees of positive effects for teachers who achieved national board certification (8-11). The studies found significant differences in student achievement for future board-certified teachers before their application to the NBPTS program, termed a "signaling effect." The results showed that these advantages persisted after certification, but the advantages over noncertified teachers were small and, in some cases, not significant. The results may have been due to selection effects: better teachers may have sought certification.

The most recent large sample study evaluated elementary and high school teachers in Florida by using a gain score analysis similar to that used in North Carolina. However, unlike the North Carolina studies, investigators found neither a prior (signaling) effect nor significant differences after certification (12). The authors concluded, "Based on our findings for Florida, the efficacy of NBPTS as a tool to improve student learning appears questionable. The 2 main potential benefits are to identify and reward productive teachers and to encourage teachers to improve their teaching skills. Our results suggest that NBPTS does neither, at least when teacher productivity is measured in terms of student achievement gains soon after a teacher becomes certified" (12).

Explicit schemes to create a pay-for-performance system, including merit pay or teacher ranking systems, have not been successful in implementation or in having consistent effects on student achievement. These results have led scholars and some educators to recommend a more direct approach, by paying teachers for how much their students learn over time.

### Growth and value-added models

State standardized tests, especially given the yearly testing requirements of NCLB, supply the necessary data to track student progress longitudinally. NCLB requires only reporting and accountability at the school or district level with cohort scores, but many states have noted that a fairer system would hold schools accountable for growth that individual students make from year to year. Although the language in this area is not always clear, I refer to change metrics as "growth scores" when they are recorded with an estimated yearly change as the basic measure; "value-added" describes a sequence of changes and projected growth patterns that are created for individual students.

Growth and value-added models address problems of selection bias for teachers. Because students may not be randomly assigned to teachers, under most state reporting systems, a teacher who attracts or is assigned lower-achieving students will be penalized if judged solely on a yearly cohort score. That lower achievement is related to student and family characteristics and perhaps prior education. Growth scores assume that the historical accumulation of these family and educational resources is captured by including the previous test in estimation models.

Controlling for prior level of achievement may not be sufficient, however, because achievement depends not only on a starting place but also a rate of growth. For example, a student who begins school at a lower level of achievement may have a steeper learning curve than a student with higher prior achievement. In this case, the yearly growth will be an invalid indicator of what was accomplished in that year. However, if a sequence of annual scores is available for each student, an average rate of progress can be determined, and we can estimate future projected achievement. This projection or trajectory can then be used as an expectation of the value added by a school or teacher over time. In this model, both the starting differences and the growth rates of students are taken into consideration, and either schools or teachers could be judged on how well students do on the basis of their projected outcomes.

Theoretically, future deviations from the trajectory (residuals in a statistical model) could be linked backward to prior teachers. This procedure would construct a value-added model for rewarding teachers. Such a model was first suggested and implemented in Tennessee (13); in recent years, a variant has been suggested as a tool for use in the teacher tenure process (14).

Implementing such a reward system at the teacher level would be associated with many problems, and integrating it into a school-level accountability system, as required by NCLB, would be even more problematic. Using value-added models to evaluate programs, which means system accountability, should be distinguished from using them to judge individual teachers. Measurement and other errors in tests are particularly problematic when the sample of students is small, as in the case of an individual teacher (15). This limitation explains a troublesome finding that teacher rankings that use value-added models are highly inconsistent from year to year (C. Koedel, unpublished data). If value added is an accurate estimate of teacher quality and effectiveness, one would expect stability over time. Measurement problems are explored in detail elsewhere (16).

Value-added methods are still the hottest topic in paying for performance in education. The approach has been used, the student-linked data records are or will be available in most states, and the method will probably be an option for states if NCLB is reauthorized in the future.

## Summary of Accountability and Performance Efforts in Education

In the past 25 years, the resources and data available to provide system-level accountability (either school or district) have improved, generating a stronger focus on student outcomes as the appropriate measure of accountability, reward, and sanctions. According to state and federal mandates, districts and schools are under pressure to increase achievement. Schools and districts are being found "in need of improvement" under NCLB — a status made available to the public. They are also facing increasing sanctions for successive years of failure.

Several conclusions can be made concerning this movement to system accountability. First, the "report card" era, applied to states, districts, and even schools, that began in the Reagan administration, has subsided. Second, NCLB, its system replacement, has been met with widespread unhappiness, and if the current administration's legislative proposals are adopted, may be essentially dismantled, eliminating in particular any punitive actions against schools.

System-level accountability has yet to be translated into successful teacher accountability, despite many efforts to install merit pay, career ladders, certification systems, and most recently, directly rewarding teachers for student success. Translating system-level accountability into individual accountability may be even more difficult for health care because of the more complex nature of the organizations and services in that field.

## From Education to Health Care

Education and health care share several characteristics when it comes to accountability and performance. Both can be examined in terms of system-level or individual accountability. As in education, system-level accountability in health care has improved in terms of measuring performance through organizational report cards, audits, and ratings of hospitals, nursing homes, and other facilities (17).

Both systems also share a hierarchy that directly affects and limits the implementation of individual-level performance incentives. Both hierarchies are flat in the sense that movement upward is generally unrelated to performance and depends primarily on credentialing and time on the job. Unlike most other public and private organizations, in education and health care, simply doing a job well will rarely allow a person to be promoted to a higher-level job. This problem cuts off the central means of reward that exists in government, the military, and the corporate world: promotion as a reward for doing a job well. The efforts in education with merit pay and career ladders can be interpreted as artificially instilling organizational advancement; unfortunately, as with most things artificial, these efforts have routinely failed.

Flat hierarchies shift the burden of reward and sanction to paying people for performance on the job. Although standard personnel practices, such as annual reviews by supervisors and peers, may be the most likely road to determining performance, union environments in both fields may make this difficult. Attempts to create performance metrics that may be more objective than supervisor judgment have been the result. In education, that led to exploring value-added assessments based on longitudinal student achievement.

The final issue is whether performance-based systems will be easier or more difficult in health care. Individual-level performance metrics will be as difficult to create and implement in health care as they were in education, if not more so. Health care hierarchies are more complex, they deal with a broader range of clients, they provide more diverse services, and they require more teamwork. In education, after all is said about joint production, school missions, and multiple stakeholders, for most of the day a single teacher is behind a closed door with students who are trying to accomplish more or less the same tasks. Although we argue that we need to judge education outcomes on more than performance on standardized tests, those tests certainly help, and the list of other performance measures mentioned is usually small.

Compare this with a routine procedure in health care, vivid in my recent memory — the colonoscopy. For a 2-hour procedure, no fewer than 11 people were involved, each performing a different function that would be evaluated on different criteria using (presumably) different metrics. To be sure, some areas of health care require fewer people and simpler tasks (such as laboratory diagnoses, routine physical examinations, immunizations), but many other areas are even more complex than a colonoscopy.

Finally, if my analysis of education is correct, with the lack of consistent success of individual-level performance accountability methods, the outlook for following those approaches in health care is even bleaker. What then to do? First, system-level performance and accountability procedures are not trivial accomplishments. This implies the need for top-down approaches of oversight and responsibility, furthering those installed in education and health care during the last decades. Second, the only reasonable approach to individual performance metrics, other than falling back on credentialing and experience as reward markers, is from the bottom up, on a unit level, with a supervisor evaluating individual employees. The complexity of the tasks, services, and patient mix in health care suggests that any overarching system would be doomed to failure. The bottom-up, unit-by-unit approach is probably being used in most instances already. Incremental tinkering and experimenting with objective measures tailored to units and jobs, with oversight by responsible supervisors, might not be a radical enough solution for many, but it still might be the right approach.
